# Scorpion Venom-Derived Peptides: A New Weapon Against Carbapenem-Resistant *Acinetobacter baumannii*

**DOI:** 10.3390/microorganisms14010068

**Published:** 2025-12-28

**Authors:** Carla Capasso, Carla Zannella, Rosa Giugliano, Annalisa Chianese, Alessandra Monti, Federica Donadio, Emanuela Esposito, Gerardo Marino, Nunzianna Doti, Anna De Filippis, Massimiliano Galdiero

**Affiliations:** 1 Department of General and Specialized Surgery for Women and Children, University of Campania “Luigi Vanvitelli”, 80138 Naples, Italy; carla.capasso@unicampania.it (C.C.); carla.zannella@unicampania.it (C.Z.); rosa.giugliano@unicampania.it (R.G.); annalisa.chianese@unicampania.it (A.C.); anna.defilippis@unicampania.it (A.D.F.); 2 Department of Veterinary Medicine and Animal Production, University of Naples Federico II, 80137 Naples, Italy; 3 Institute of Biostructures and Bioimaging (IBB), National Research Council (CNR), 80131 Naples, Italy; alessandra.monti@ibb.cnr.it (A.M.); nunzianna.doti@cnr.it (N.D.); 4 Institute of Applied Sciences and Intelligent Systems (ISASI), Naples Cryo Electron Microscopy Laboratory—EYE LAB, National Research Council (CNR), Via Pietro Castellino 111, 80131 Naples, Italy; federica.donadio@na.isasi.cnr.it (F.D.); emanuela.esposito@cnr.it (E.E.); 5 UOC Food Hygiene and Nutrition Service, 84124 Salerno, Italy; gerarm@libero.it; 6 UOC Virology and Microbiology, University Hospital “Luigi Vanvitelli”, 80138 Naples, Italy

**Keywords:** *Acinetobacter baumannii*, antimicrobial peptides, scorpion venom-derived peptides, carbapenem-resistant bacteria, biofilm, antibacterial activity

## Abstract

*Acinetobacter baumannii* (*A. baumannii*) is an opportunistic pathogen associated with healthcare-related infections and is of particular concern due to its high level of antibiotic resistance and its ability to form biofilms. The global emergence of carbapenem-resistant *A. baumannii* highlights the urgent need for alternative therapeutic strategies. This study investigated the antibacterial and antibiofilm activities of two scorpion venom-derived peptides, pantinin-1 and pantinin-2, against a reference strain and a clinical isolate of *A. baumannii*. We found that both peptides, in the non-cytotoxic concentration range, have strong bactericidal activity, showing a minimum inhibitory concentration (MIC) of 6.25 μM and 12.5 μM for pantinin 1 and 2, respectively. Scanning electron microscopy (SEM) analysis showed that the peptides cause extensive damage to the bacterial membrane. Furthermore, both peptides showed potent antibiofilm activity, inhibiting adhesion and maturation, arresting biofilm expansion, and reducing the expression of key biofilm-associated genes (*bap*, *pgaA*, and *smpA*). Altogether, these findings indicate that pantinin-1 and pantinin-2 act through a dual mechanism, combining bactericidal and antivirulence activities. Their strong efficacy at low micromolar concentrations, together with low cytotoxicity, underscores their potential as innovative therapeutic candidates against infections caused by carbapenem-resistant, biofilm-forming *A. baumannii*.

## 1. Introduction

The emergence of antibiotic resistance poses a major global health threat, undermining the ability to treat both common and complicated infections and compromising advancements in modern medicine [1,2,3]. Within this context, the ESKAPE pathogens (*Enterococcus faecium*, *Staphylococcus aureus*, *Klebsiella pneumoniae*, *Acinetobacter baumannii*, *Pseudomonas aeruginosa*, and *Enterobacter* spp.) have been prioritized by the World Health Organization and other public health agencies due to their capacity to rapidly acquire and disseminate multiple resistance mechanisms, thereby evading most currently available antibiotics [4,5].

Among Gram-negative bacteria, *Acinetobacter baumannii* (*A. baumannii*) stands out as a particularly challenging pathogen because of its diverse virulence and resistance patterns. These include a double membrane that restricts antibiotic penetration, highly efficient efflux pumps, the production of antibiotic-degrading enzymes, and a remarkable ability to acquire and spread resistance genes via mobile genetic elements [6,7]. In addition, *A. baumannii* demonstrates exceptional resilience to harsh environmental conditions, enabling survival on hospital surfaces and medical equipment. This persistence underlines its role as a major cause of healthcare-associated infections, including ventilator-associated pneumonia, bloodstream infections, and wound infections [3,8]. Resistance rates of *A. baumannii* to key antibiotic classes, including carbapenems, cephalosporins, fluoroquinolones, and aminoglycosides, now exceed 90% [3,9]. Alarmingly, resistance has also emerged against last-line agents such as colistin, leaving very limited therapeutic options and contributing to elevated morbidity and mortality among affected patients. This problem extends beyond traditional antibiotics: even newly developed macrocyclic and peptide antibiotics, once considered effective and relatively safe, have rapidly encountered the emergence of resistant strains [5,10,11]. The ability of *A. baumannii* to form biofilms is a key virulence factor that greatly contributes to its persistence and resistance in clinical settings, enabling long-term adhesion to surfaces and medical equipment. Biofilms also impair host immune defenses and reduce the efficacy of antimicrobial agents, leading to infections that are difficult to eradicate and may progress to chronic conditions. Consequently, treating infections caused by biofilm-forming *A. baumannii* in healthcare environments remains particularly challenging [12].

Therefore, searching for alternative therapeutic strategies has become a global priority, and antimicrobial peptides (AMPs) have emerged as promising candidates [13]. AMPs are naturally occurring or synthesized molecules, typically short chains of 5–40 amino acid residues. They are generally cationic and amphipathic, properties that enable selective interaction with microbial membranes. This interaction can disrupt membrane integrity through destabilization and pore formation, ultimately leading to cell death [14,15,16]. Beyond their direct membranolytic activity, some AMPs interfere with intracellular processes, such as the recently identified translation initiation factor 1 (IF1)-derived peptide from *Clostridium difficile*, which inhibits bacterial growth by binding to the 30S ribosomal subunit [17]. Additionally, AMPs can also modulate host immune responses and act synergistically with conventional antibiotics, thereby reducing the likelihood of resistance development [18].

Numerous AMPs have been identified across diverse sources, including humans, insects, amphibians, and microorganisms [19]. Among AMPs of animal origin, the pantinin family, derived from the venom of the scorpion *Pandinus imperator*, has attracted considerable interest. Specifically, pantinin-1 (primary sequence: GILGKLWEGFKSIV-NH_2_) and pantinin-2 (primary sequence: IFGAIWKGISSLL-NH_2_) are non-disulfide-bridged peptides with 14- and 13-amino acids, respectively, and α-helical structure, with antimicrobial activity against Gram-positive bacteria, fungi, and viruses [20,21,22,23]. Recent studies have also shown that pantinins are effective against multi-resistant strains of *Klebsiella pneumoniae* (*K. pneumoniae*), acting rapidly on the bacterial outer membrane, reducing the expression of virulence genes, and exhibiting a low propensity to induce resistance [23]. In addition, these peptides display good stability under physiological conditions and low cytotoxicity toward eukaryotic cells, making them promising candidates for the development of novel antimicrobial therapies [24].

Despite these interesting results, the activity of pantinin-1 and pantinin-2 against particularly problematic Gram-negative bacteria such as *A. baumannii* remains underexplored. Considering the critical role of *A. baumannii* in nosocomial infections and its remarkable capacity to develop multiple resistance mechanisms, it is essential to assess whether these peptides serve as effective alternatives or adjuncts to conventional antibiotics against this pathogen [25]. In the present study, we investigated the antimicrobial activities of pantinin-1 and pantinin-2 against *A. baumanni*. In detail, we evaluated their effect on biofilm formation and maturation, as well as their potential to interfere with key resistance mechanisms. Through these analyses, we aim to gain a deeper understanding of the therapeutic potential of pantinins as novel treatment options for infections caused by multidrug-resistant ESKAPE pathogens, thereby contributing new insights to the fight against antibiotic resistance.

## 2. Materials and Methods

### 2.1. Peptide Synthesis and Characterization

Peptides were synthesized with an amidation at the C-terminal end using the solid-phase Fmoc (fluorenylmethyloxycarbonyl) strategy on the SYRO I automated peptide synthesizer (Biotage, Uppsala, Sweden), following well-established protocols [23]. Briefly, after synthesis, peptides were purified via reversed-phase high-performance liquid chromatography (RP-HPLC) on a WATERS 2545 preparative system equipped with a UV/Vis detector (Waters 2489, Waters, Milford, MA, USA). The identity and purity of the peptides were confirmed through liquid chromatography-mass spectrometry (LC-MS) analysis using a LTQ XL™ linear ion trap mass spectrometer (Thermo Scientific™, Waltham, MA, USA). Separation was performed on a Waters xBridge C18 column (5 μm, 2.1 × 50 mm) with a linear gradient of acetonitrile (CH_3_CN) and 0.05% trifluoroacetic acid (TFA) in water and TFA, ranging from 10% to 80% acetonitrile over 10 min at a flow rate of 0.2 mL/min. The peptide exhibited a purity exceeding 95%. Peptide concentration was determined by UV-Vis spectroscopy at 280 nm using a standard 1-cm path length cuvette. Concentrations were calculated via the Beer-Lambert Law (A = Ɛ × l × C) using a molar extinction coefficient (Ɛ) of 5500 M^−1^ cm^−1^. After baseline correction with the appropriate buffer, samples were measured in duplicate and results expressed as the mean value.

### 2.2. Bacterial Strains

To assess the antimicrobial activity of pantinins, we used a standard strain of *A. baumannii* (BAA-747, acquired from the American Type Culture Collection, ATCC, Manassas, VA, USA) and a clinical strain kindly provided by the Complex Operative Unit of Virology and Microbiology of the University Hospital “Luigi Vanvitelli” (Naples, Italy). The *A. baumannii* clinical strain was isolated on MacConkey agar (Oxoid, Basingstoke, Hampshire, UK) and incubated overnight at 37 °C. Bacterial identification and antimicrobial susceptibility testing were performed using the Matrix-Assisted Laser Desorption/Ionization-Time-of-Flight system (MALDI-TOF, Bruker Daltonics, Bremen, Germany) and the Phoenix BD system (Becton Dickinson, Franklin Lakes, NJ, USA). After 16 h of incubation, resistance patterns were interpreted according to EUCAST breakpoints. Table S1 (Supplemental Material) reports the antibiogram profile of the same strain.

Before antibacterial testing, both strains were subcultured on Müller-Hinton agar (MHA, Sigma-Aldrich, St. Louis, MO, USA) and grown in Müller-Hinton broth. For biofilm assays, *A. baumannii* strains were streaked on Luria–Bertani (LB) agar plates (Sigma-Aldrich) and grown in LB broth supplemented with 1% glucose (Sigma-Aldrich) at 37 °C under aerobic conditions [26].

### 2.3. Minimum Inhibitory Concentration (MIC_80_) and Minimal Bactericidal Concentration (MBC) Determination

The antibacterial activity of pantinin-1 and pantinin-2 was evaluated against both standard and clinical strains of *A. baumannii*. The minimum concentration required to inhibit 80% bacterial growth (MIC_80_) and the minimum bactericidal concentration (MBC), i.e., the lowest peptide concentration that resulted in a 99.9% killing of bacterial cells, were determined using the broth microdilution method [27]. Peptide activity was tested over a concentration range of 50–1.56 μM.

Briefly, a pure colony grown in MHA was inoculated into MH broth and incubated at 37 °C with shaking at 180 rpm until the mid-logarithmic growth phase was reached. A bacterial suspension of 1 × 10^6^ colony-forming units (CFU)/mL in fresh MH broth was then prepared and dispensed (50 μL/well) into a microtiter plate preloaded with serial two-fold dilutions of pantinin-1 or pantinin-2, resulting in a final inoculum of 5 × 10^5^ CFU/well. Gentamicin (4 μg/mL) was used as a positive control against the *A. baumannii* ATCC strain, while colistin (5 μg/mL) was used as a positive control against the clinical isolate strain. Untreated bacterial cells served as a negative control. After 20 h of incubation at 37 °C, bacterial turbidity was measured spectrophotometrically at 600 nm. Growth inhibition was calculated using the following formula:

% *Growth inhibition* = [1 − (*Abs*_600nm_
*test sample*)/*Abs*_600nm_
*CTR negative*] × 100

To determine MBC values, 50 μL from wells showing no visible growth were spread onto MHA plates, as described previously [28]. Plates were incubated for 24 h at 37 °C, and bacteria were counted and enumerated. The MBC was defined as the lowest peptide concentration that resulted in a 99.9% killing of bacterial cells.

#### Time-Kill Kinetic Assay

Time-kill kinetic assays were performed to monitor the activity of pantinin-1 and pantinin-2 over time against the planktonic form of *A. baumannii* BAA-747, using the same experimental conditions as the MIC assay [29]. At selected time points (0, 1, 2, 4, 6, and 18 h), 100 μL aliquots from each treatment group (½× MIC, 1× MIC, and 2× MIC) were serially diluted 10-fold in 1× PBS, plated onto MHA, and incubated overnight at 37 °C. Bacterial colonies were finally counted, and the CFU/mL was calculated. Peptide-free cultures served as negative controls, while gentamicin (4 μg/mL) was included as a positive control.

### 2.4. Synergism Assay

To evaluate the combined effect of pantinin-1 and pantinin-2, a synergism assay was performed [30,31]. An *A. baumannii* inoculum of 2 × 10^5^ CFU/mL was prepared for the test. The IC_50_ of each peptide was selected, and 10 μL of the corresponding peptide solutions were added to the wells of a 96-well microtiter plate, either individually or in combination. The plate was then incubated at 37 °C for 24 h. Bacterial growth inhibition was determined spectrophotometrically at 600 nm and calculated using the following formula:

% *Growth inhibition* = [1 − (*Abs*_600nm_
*test sample*)/*Abs*_600nm_
*CTR negative*] × 100

### 2.5. Antibiofilm Activity

#### 2.5.1. Biofilm Adhesion and Maturation

The efficacy of synthetic peptides pantinin-1 and pantinin-2 in counteracting the biofilm adhesion (2 h) and formation (24 h) phases of the *A. baumannii* ATCC strain was evaluated [32]. An *A. baumannii* inoculum was prepared at a density of 2 × 10^8^ CFU/mL in LB broth supplemented with 1% glucose and exposed to the peptides at concentrations ranging from 1.56 to 25 μM. Treatments were performed for 2 h and 24 h at 37 °C under static conditions. Untreated bacterial cultures served as negative controls. Following incubation, the biofilms were washed twice with 1× PBS to remove non-adherent cells and stained with 0.05% crystal violet (CV) for 40 min. Excess dye was discarded, and the wells were rinsed with 1× PBS. The bound CV was then solubilized with 100% ethanol for 10 min. In parallel, biofilm metabolic activity was assessed using the 3-(4,5-dimethylthiazol-2-yl)-2,5-diphenyltetrazolium bromide (MTT, Sigma-Aldrich) assay under the same experimental conditions. After 2 h and 24 h of peptide exposure, biofilms were washed and incubated with 0.5 mg/mL MTT solution for 3 h at 37 °C. The resulting formazan crystals were dissolved in 100% DMSO for 10 min. For both assays, absorbance was measured at 570 nm. Biofilm inhibition and metabolic activity were calculated according to the following formulas:

% *Biofilm inhibition* = 1 − (*OD*_570 nm_
*test sample*/*OD*_570 nm_ *CTR negative*) × 100

% *Metabolic activity* = 1 − (*OD*_570 nm_ *test sample*/*OD*_570 nm_ *CTR negative*) × 100

#### 2.5.2. Biofilm Degradation

Biofilm eradication activity was quantified using the CV colorimetric assay [33]. An overnight culture of *A. baumannii* ATCC was adjusted to a density of 2 × 10^8^ CFU/mL in LB broth supplemented with 1% glucose and added to each well of a 96-well microtiter plate. Plates were incubated at 37 °C for 48 h under static conditions to allow mature biofilm formation. After incubation, planktonic cells were removed, and the biofilms were washed twice with 1× PBS. The preformed biofilms were treated with the peptide concentrations described above for 24 h at 37 °C. The supernatant was discarded after treatment, and the biofilms were washed and stained with 0.05% CV. The bound dye was solubilized with DMSO, and absorbance was measured at 570 nm. Biofilm degradation was calculated according to the following formula:

% *Biofilm degradation* = 1 − (*OD*_570 nm_ *test sample*/*OD*_570 nm_ *CTR negative*) × 100

### 2.6. Scanning Electron Microscopy (SEM) Analysis

The morphological changes induced by pantinin-1 and pantinin-2 were studied using SEM [23,28]. Briefly, bacterial cultures were grown to the exponential phase and harvested by centrifugation at 1000× *g* for 10 min. The resulting pellets were resuspended in 10 mM PBS to an OD600 of 0.2. The bacterial suspensions were then exposed to peptides at different concentrations (MIC, 1× MIC, and ½× MIC) and incubated for 60 min at 37 °C. After treatment, cells were collected by centrifugation, rinsed with PBS, and fixed overnight at 4 °C in 2.5% (*v*/*v*) glutaraldehyde. Finally, samples were washed with PBS and dehydrated through a graded ethanol series (50, 70, 90, and 100% *v*/*v*) for 15 min at each step. Images were acquired using a ThermoFisher Scientific Aquilos 2 double-beam FIB-SEM. The acquisition parameters were current 13 pA, voltage 2 kV, working distance 4.6 mm, field of view 20.7 μm, stage tilt 0°, and magnification 5.000×.

### 2.7. Molecular Analysis

PCR assays were performed to evaluate the expression of three virulence-associated genes, *smpA*, *bap*, and *pgaA*, using specific primers purchased from Eurofins (Vimodrone, Milan, Italy), as listed in Table 1.

Gene expression was evaluated following biofilm formation under different experimental conditions, as described above. Treatments were stopped after 2 h, 24 h, and 72 h during the attachment, inhibition, and degradation assays, respectively. Total RNA was extracted from the collected bacterial pellets using TRIzol^®^ reagent (Thermo Fisher Scientific, Waltham, MA, USA). For each experimental condition, triplicate samples were pooled before extraction to ensure sufficient RNA yield. The protocol included centrifugation of the lysates, followed by phase separation with chloroform, RNA precipitation with isopropanol, and washing with ethanol. The purified RNA pellet was finally resuspended in nuclease-free water and quantified using a Nanodrop spectrophotometer (Nanodrop 2000, Thermo Fisher Scientific) to assess concentration and purity [34]. Subsequently, 1 μg of total RNA was reverse-transcribed into cDNA using the RT All-in-One MasterMix (Applied Biological Materials, Richmond, BC, Canada). PCR reactions for each target gene were performed in a total volume of 25 µL, containing 1 µL of cDNA, 4 µL of FIREPol Master Mix (Solis Biodyne, Tartu, Estonia), 0.5 µL of each primer, and 13 µL of distilled water. Amplified DNA fragments were resolved by horizontal electrophoresis on 2% agarose gels at 120 V for 35 min. Samples were mixed with DNA Loading Dye (Microtech, Naples, Italy), and molecular sizes were determined using the 1 kb Opti-DNA Marker (Microtech). The biofilm of *A. baumannii* ATCC BAA-747 served as a positive control.

### 2.8. Statistical Analysis

All experiments were performed in three biological and three technical replicates. Data are expressed as the mean ± Standard Deviation (SD). Statistical significance was assessed using One-way ANOVA or Two-way ANOVA, followed by Dunnett’s post hoc test for multiple comparisons. A *p*-value < 0.05 was considered statistically significant. Graphs were generated using GraphPad Prism version 9.5.1 for macOS (GraphPad Software, San Diego, CA, USA).

## 3. Results

### 3.1. Evaluation of Antibacterial Activity

**Figure 1:**
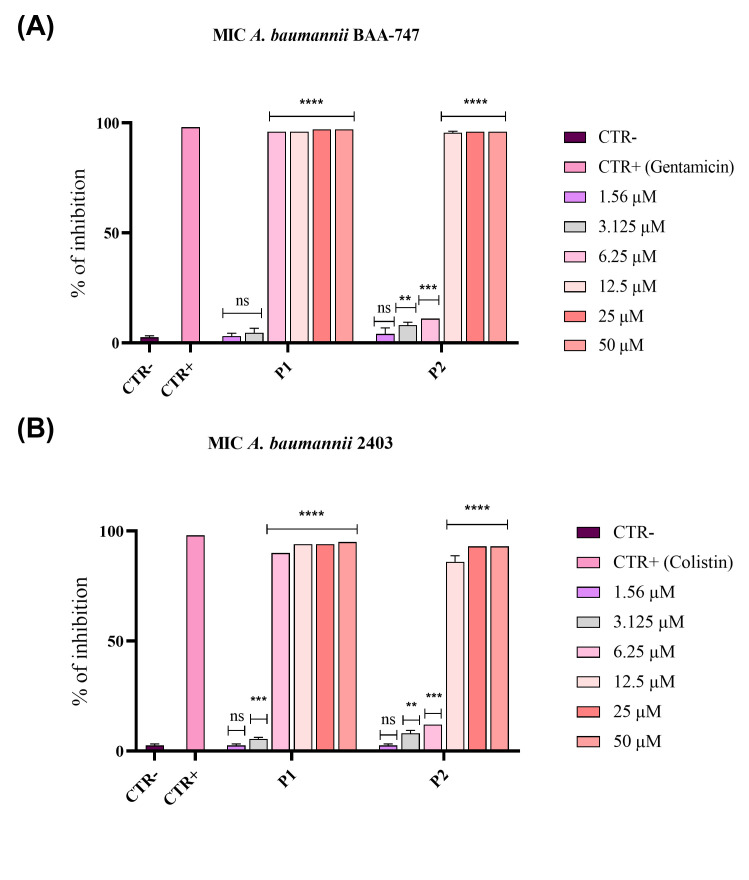
Inhibition of *A. baumannii* growth by pantinin-1 and pantinin-2 through the broth microdilution method. Bacterial cells from an ATCC strain BAA-747 (**A**) and the clinical strain 2403 (**B**) were pantinin-1 (P1), pantinin-2 (P2), gentamicin, or colistin (CTR+). Untreated cells represented the negative control (CTR−). Data represent the mean ± SD. Dunnett’s multiple comparison test: (**A**) **** *p*-value < 0.001; *** *p*-value = 0.0005; ** *p*-value = 0.0077 and ns = not significant; (**B**) **** *p*-value < 0.0001; *** *p*-value = 0.0002; ** *p*-value < 0.0077 and ns = not significant.

The antibacterial activity of pantinins was evaluated by the broth microdilution method and kinetic time-kill assays (see Section 2 for details) against bacterial cells from an ATCC strain BAA-747(A) and clinical strain 2403. Broth dilution tests showed that both synthetic peptides exhibited antibacterial activity against *A. baumannii* in a dose-dependent manner (Figure 1).

Pantinin-1 exhibited the strongest antibacterial activity against ATCC and clinical strains, effectively inhibiting bacterial growth at an MIC_80_ of 6.25 μM (indicated as P1 in Figure 1A,B). On the other hand, pantinin-2 achieved complete inhibition at 12.5 μM against both strains (indicated as P2 in Figure 1A,B). Moreover, to distinguish between bacteriostatic and bactericidal effects of pantinins, the MBC was determined for both strains. Both peptides exhibited bactericidal activity at their respective MIC_80_ values.

**Figure 2:**
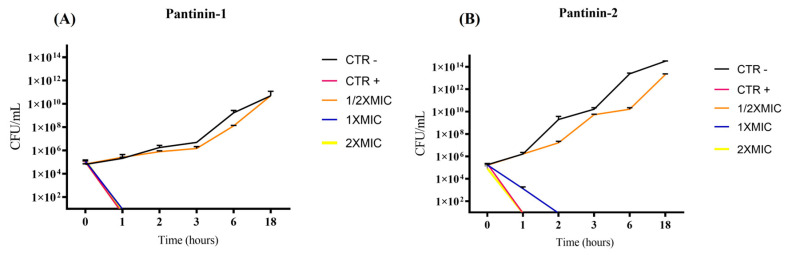
Time-kill kinetic assay of pantinins against *A. baumannii* ATCC. Temporal killing profiles of pantinin-1 (**A**) and pantinin-2 (**B**) are shown. CTR−: untreated bacteria; CTR+: bacteria treated with gentamicin (4 μg/mL).

The antibacterial activity was further characterized through time–kill kinetic assays, which analyzed the growth of *A. baumannii* BAA-747 over time following the treatment with each peptide (Figure 2).

Pantinin-1 completely eradicated bacterial cells within 1 h at both 2× MIC (12.5 μM) and MIC (6.25 μM) (Figure 2A). In contrast, pantinin-2 required 2 h to achieve complete eradication at 2× MIC (25 μM) and MIC (12.5 μM) (Figure 2B).

### 3.2. Effect of Pantinins on Different Stages of Biofilm Formation

**Figure 3:**
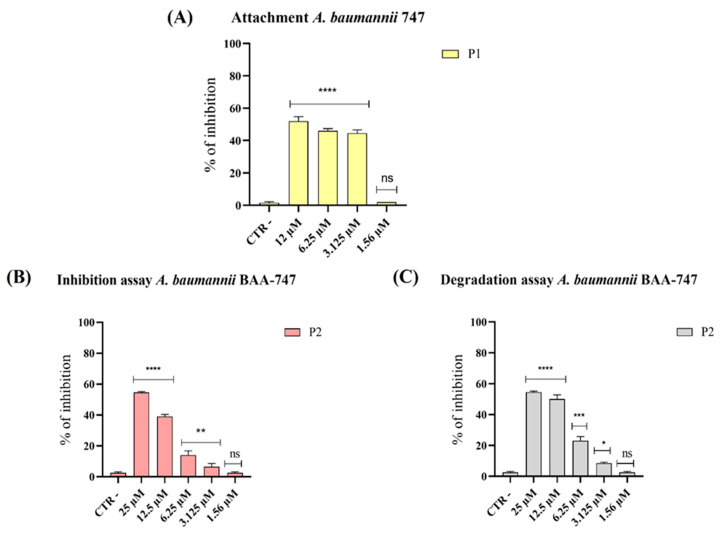
Evaluation of anti-biofilm activity of pantinins against *A. baumannii*. (**A**) Impact of pantinin-1 (P1) on *A. baumannii* biofilm in the early adhesion stages; (**B**) effect of pantinin-2 (P2) during biofilm formation; and (**C**) activity against preformed biofilms. CTR−: untreated bacteria. Data represent the mean ± SD. Statistical significance was determined using Dunnett’s multiple comparison test: (**A**) **** *p*-value < 0.0001 and ns: not significant; (**B**) **** *p*-value < 0.0001, ** *p*-value = 0.0015 and ns = not significant; (**C**) **** *p*-value < 0.0001, *** *p*-value = 0.0001, * *p*-value = 0.457 and ns: not significant.

Biofilm formation provides bacteria with protection against the host immune system and increases their resistance to antibiotics by 10- to 1000-fold compared with their planktonic form [35]. In the present study, biofilms were exposed to peptide concentrations ranging from 2× MIC to 1.56 µM. Biomass biofilm was quantified using the CV staining method, while the viability of sessile cells was assessed using the MTT assay (Figure 3).

Our results showed a significant reduction in biofilm biomass following treatment with pantinin-1 during the initial adhesion phase (Figure 3A) and with pantinin-2 during the maturation phase (Figure 3B). Exposure to pantinin-1 impaired the adhesion capacity of *A. baumannii* by 50% and 45% at concentrations of 12.5 µM and 6.25 µM, respectively (Figure 3A). Conversely, pantinin-2 exhibited notable activity during the biofilm maturation phase, reducing biofilm biomass by 50% and 40% at concentrations of 25 µM and 12.5 µM, respectively (Figure 2B). Furthermore, analysis of mature biofilms revealed that pantinin-2 was effective in disrupting preformed biofilms (Figure 3C). Specifically, treatment with pantinin-2 reduced biofilm biomass by 55% at 25 μM and by 50% at 12.5 μM.

### 3.3. Evaluation of Virulence Gene Expression

**Figure 4:**
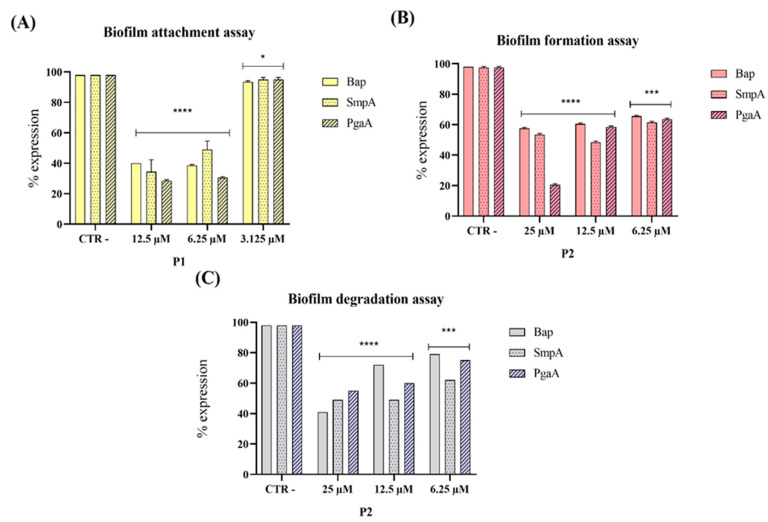
Gene expression levels of the three genes *bap*, *smpA,* and *pgaA* by PCR analysis after treatment of pantinin-1 (P1) and pantinin-2 (P2). Different assays were conducted: (**A**) biofilm attachment assay; (**B**) biofilm formation assay; and (**C**) biofilm degradation assay. Data represent the mean ± SD. Statistical significance was determined using Dunnett’s multiple comparison test: (**A**) **** *p*-value < 0.0001 and * *p*-value = 0.0299; (**B**) **** *p*-value < 0.0001 and *** *p*-value = 0.0001; (**C**) **** *p*-value < 0.0001 and *** *p*-value = 0.0001.

Several key factors contribute to the virulence of *A. baumannii*, including biofilm production, the synthesis of poly-β-(1-6)-N-acetylglucosamine polysaccharide (PNAG), and cell adhesion. To evaluate the ability of pantinin-1 and pantinin-2 to suppress these virulence determinants, we analyzed the expression of three specific genes (Figure 4): *bap*, involved in biofilm formation [36]; *pgaA*, which encodes proteins responsible for PNAG synthesis [37], and *smpA*, an outer membrane assembly protein that contributes to the structural integrity of the bacterium [38].

Our results demonstrated that both pantinin-1 and pantinin-2 significantly downregulated the expression of the virulence genes analyzed. In particular, pantinin-1 reduced gene expression during the initial attachment phase (Figure 4A), whereas pantinin-2 decreased expression in both the biofilm formation inhibition assay (Figure 4B) and mature biofilm degradation assay (Figure 4C). These findings highlight the ability of pantinins to interfere with biofilm development by targeting key virulence factors essential for biofilm formation and stability.

### 3.4. The Effects of Pantinin-1 and Pantinin-2 on the Integrity of A. baumannii Surface

**Figure 5:**
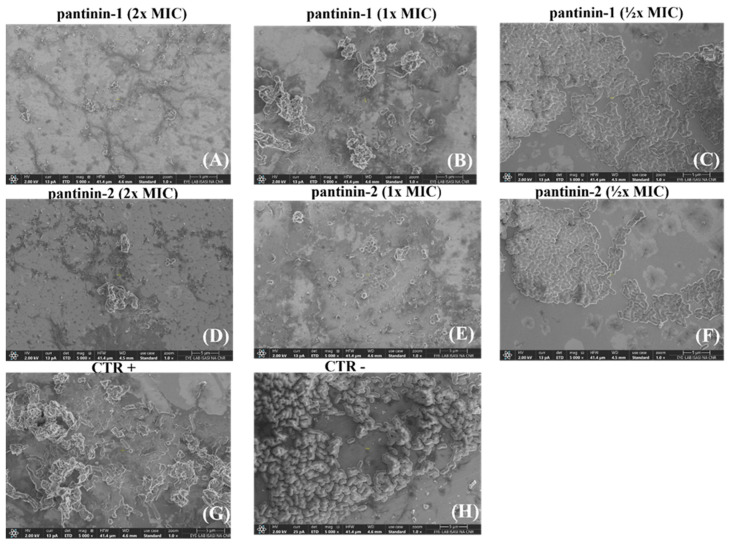
SEM analysis of *A. baumannii* cells treated with different concentrations of pantinins. Cells were exposed to 2× MIC (**A**,**D**), 1× MIC (**B**,**E**), and ½× MIC (**C**,**F**) of each peptide. CTR− corresponds to untreated bacteria (**H**), while CTR+ corresponds to bacteria treated with gentamicin (4 μg/mL) (**G**). Images were acquired at a magnification of 5000×.

We investigated the effects of pantinins on the surface integrity of the *A. baumannii* surface using Scanning Electron Microscopy (SEM) (Figure 5).

Bacterial cells were treated with different concentrations of pantinin-1 and pantinin-2 (½× MIC, 1× MIC, and 2× MIC), and surface morphological alterations were examined. Compared with the negative control (untreated bacterial cells), peptide-treated cells exhibited markedly rougher surfaces, along with visible cell shrinkage and the presence of extracellular debris, likely resulting from leakage of intracellular material due to membrane damage. Samples treated with 2× MICs (Figure 5A,D) displayed more pronounced morphological alterations than those treated with MICs (Figure 5B,E), indicating that higher peptide concentrations cause more extensive damage to *A. baumannii* cells. In contrast, the negative control (Figure 5H) showed smooth, intact cell surfaces with a typical bacillary morphology.

### 3.5. Synergistic Effect of Pantinins Against A. Baumannii

**Figure 6:**
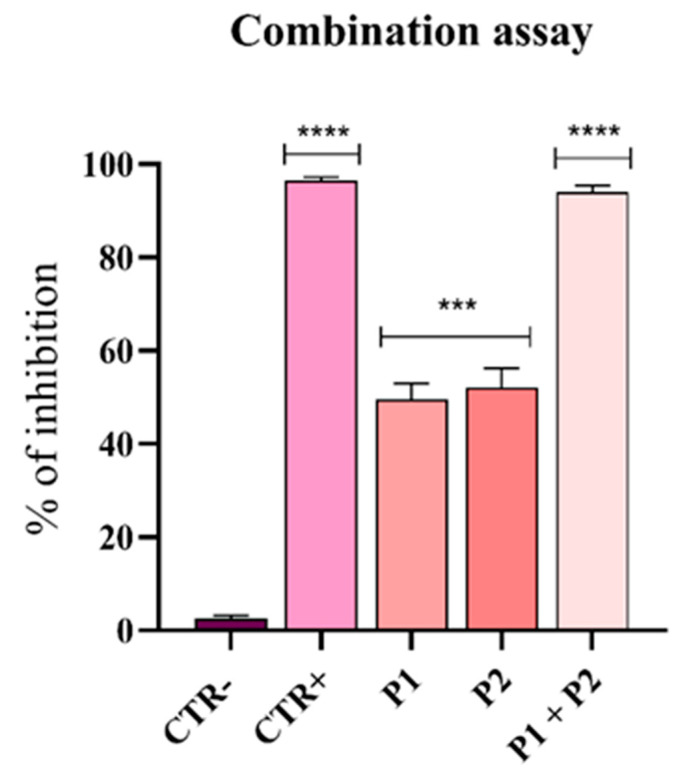
Synergistic effect of pantinin-1 and pantinin-2. Data are presented as mean ± SD. CTR−: untreated cells; CTR+: cells treated with gentamicin (4 μg/mL). Statistical analysis was performed using two-way ANOVA followed by Dunnett’s multiple comparison test. Significance values refer to treated cells. **** *p*-value < 0.0001; *** *p*-value = 0.0003.

In severe bacterial infections, combination therapy is often more effective than monotherapy in eradicating pathogens. The combined antibacterial activity of pantinin-1 and pantinin-2 was evaluated to assess potential synergy (Figure 6).

As previously determined, the MIC_80_ of pantinin-1 against *A. baumannii* was 6.25 µM (Figure 1A), while the concentration at which we observed a 50% inhibition (IC_50_) was 3.2 µM. For pantinin-2, the MIC_80_ was 12.5 µM (Figure 1B), while its IC_50_ was 9.7 µM. The combination assay revealed that when both peptides were applied at their IC_50_ simultaneously, the antibacterial effect was strongly improved with respect to that observed with each peptide applied individually at its MIC (Figure 1).

## 4. Discussion

*A. baumannii* remains a major challenge in healthcare settings due to its ability to persist in hospital environments, form resilient biofilms, and acquire multiple resistance determinants. The increasing prevalence of MDR and extensively drug-resistant (XDR) strains, including those resistant to last-line antibiotics such as colistin, underscores the urgent need for alternative therapeutic strategies [27,28,29,30,31]. In this context, AMPs have emerged as promising alternative or adjunct therapies. Cationic α-helical antimicrobial peptides (AMPs), such as those derived from scorpion venom, often exert their antibacterial effects by disrupting bacterial membranes, which reduces the likelihood of bacteria developing classical resistance mechanisms [14].

Our findings demonstrate that pantinin-1 and pantinin-2 can effectively reduce bacterial growth and disrupt biofilms, suggesting that these peptides may offer a promising approach to counteract infections caused by resistant *A. baumannii* strains.

We observed a strong antibacterial effect of pantinins against the *A. baumannii* strain and the clinical isolate CRAB. Specifically, pantinin-1 showed the greatest potency, with a MIC of 6.25 μM; while pantinin-2 exhibited a MIC of 12.5 μM, against both microbes (Figure 1). Notably, these values also corresponded to the MBCs, indicating that both peptides exerted a bactericidal rather than bacteriostatic effect. The antibacterial action of both peptides was rapid, as demonstrated by the time-kill kinetic assay (Figure 2). Pantinin-1 completely eradicated bacterial cells within 1 h (Figure 2B), showing an effect comparable to that of gentamicin used as a positive control. In contrast, pantinin-2 exhibited a slower bactericidal activity, with inhibition of bacterial growth observed after 2 h of treatment. These findings are consistent with our previously reported data on the antibacterial potential of pantinins against *K. pneumoniae* [18]. In that study, pantinin-1 and pantinin-2 exhibited rapid bactericidal activity within 1 h, with MIC_80_ values ranging from 6 to 25 µM for standard strains and from 25 to 50 µM for carbapenemase-producing clinical isolates. Importantly, while pantinin-1 exhibited greater potency against bacteria, pantinin-2 exerted a stronger efficacy against viruses [21,22], evidencing a different pattern of action. Such differences in efficacy may arise from variations in amino acid sequence or peptide conformation, potentially influencing their binding affinity to the pathogen membrane.

The antimicrobial activity of pantinin-1 and pantinin-2 is primarily mediated through a membranolytic mechanism, which involves direct interactions with bacterial outer membrane lipopolysaccharides (LPS). Circular dichroism (CD) spectroscopy revealed that both pantinins adopt an α-helical conformation upon contact with LPS, supporting their membrane-targeting activity [18]. However, pantinin-2 exhibited greater hydrophobicity and a more pronounced amphipathic character, with a higher intrinsic propensity to adopt a helical conformation, as demonstrated through nuclear magnetic resonance (NMR) [22], which may underlie its enhanced antiviral activity. In contrast, pantinin-1 appears to be more effective against bacteria, possibly reflecting differences in how these peptides interact with distinct membrane architectures. Indeed, viral and bacterial membranes differ markedly in lipid composition and curvature, which may preferentially favor the membrane insertion and activity of pantinin-2 or pantinin-1, respectively.

SEM analysis suggested a membranolytic effect, as bacterial cell membranes appeared with surface roughness and morphological changes (Figure 5). At MIC_80_ values, only a small fraction of intact cells remained, whereas most bacteria displayed pronounced morphological alterations, including membrane roughness, deformation, and loss of structural integrity. At 2× MIC, SEM images revealed extensive cellular disruption and debris, consistent with severe membrane destabilization, cytoplasmic leakage, and eventual cell lysis. The cationic nature of pantinins facilitates strong electrostatic interactions with negatively charged phospholipids in bacterial membranes, promoting peptide insertion and rapid membrane disruption, which explains their rapid bactericidal activity [33]. In contrast, eukaryotic cell membranes, enriched in neutral or zwitterionic lipids and cholesterol, are less susceptible to peptide incorporation, explaining the higher concentrations required to affect host cells [35]. These findings align with recent reports; for example, Kumar et al. reviewed natural AMPs and synthetic analogs targeting *A. baumannii* membranes and cell walls, highlighting membrane disruption as an effective mechanism that is less prone to resistance development [39]. Overall, these results underscore that electrostatic targeting of bacterial membranes is central to the antimicrobial activity of cationic peptides and demonstrate their potent ability to compromise the cell membrane integrity of MDR pathogens such as *A. baumannii*.

Biofilm formation is a major determinant of antimicrobial resistance, as bacteria within biofilms can exhibit resistance levels hundreds to thousands of times higher than their planktonic counterparts [36]. In this study, pantinin-1 and pantinin-2 effectively interfered with multiple stages of biofilm development (Figure 3). Pantinin-1 inhibited the initial adhesion phase by 45% at 6.25 µM (Figure 3A), while pantinin-2 reduced biofilm formation by 55% at 25 µM and decreased mature biofilm biomass by 50% at 12.5 µM (Figure 3B,C). Both peptides also downregulated the expression of key virulence-associated genes (Figure 4), including *bap*, *pgaA*, and *smpA*, indicating that their activity extends beyond direct bactericidal effects to the suppression of biofilm formation, extracellular polysaccharide production, and membrane integrity. This suggests a multifaceted mechanism combining bactericidal and anti-virulence effects, which may help reduce bacterial pathogenicity and limit the development of resistance. This secondary mechanism, involving biofilm targeting and downregulation of virulence genes, is shared by other AMPs, such as human β-defensin 3 [40,41]. This peptide inhibits biofilm formation by reducing the expression of biofilm-associated genes *icaA* and *icaD*, while increasing the expression of *icaR*. Similarly, α-mangostin downregulated genes related to persister cell and biofilm formation, including *norA*, *norB*, *dnaK*, *groE*, and *mepR* [42].

The antibiofilm properties of scorpion venom–derived peptides remain largely underexplored; for instance, Hp1404, isolated from *Heterometrus petersii* venom, showed potent antibiofilm activity against *Pseudomonas aeruginosa*, significantly reducing biofilm formation and effectively eradicating mature biofilms at low micromolar concentrations [37]. These findings collectively highlight the considerable potential of scorpion peptides as innovative therapeutics to combat biofilm-associated infections caused by Gram-negative pathogens, and specifically by *A. baumannii*, paving the way for future preclinical and clinical investigations of these compounds. We have previously demonstrated that pantinin-1 and pantinin-2 exhibit high stability in human serum and low cytotoxicity toward mammalian cells [21,22,23]. Indeed, the stability of pantinins was previously assessed by incubation in serum for up to 16 h [23]. Pantinin-1 remained largely stable throughout this period, whereas pantinin-2 started to degrade after 4 h. Additionally, the cytotoxicity of both pantinins was evaluated using the 3-(4,5-dimethylthiazol-2-yl)-2,5-diphenyltetrazolium bromide (MTT) assay in human keratinocytes (HaCaT) [23] and glioma (U-87 MG) cells [21], with significant toxicity observed only for pantinin-2 at 100 μM. In the present study, we further show that the two peptides can be combined, resulting in an enhanced antibacterial effect (Figure 6). Combining the peptides is particularly relevant because it may allow for synergistic interactions that reduce the effective concentrations needed for bacterial eradication, limit the likelihood of resistance development, and broaden the spectrum of activity against MDR strains. This strategy could thus improve therapeutic efficacy while minimizing potential toxicity, highlighting the potential of pantinin combinations as a novel approach to treat infections caused by highly resistant pathogens such as *A. baumannii*.

## 5. Conclusions

This study highlights the potent antimicrobial and antibiofilm activities of pantinin-1 and pantinin-2, two α-helical peptides derived from *Pandinus imperator* venom, against CRAB pathogens. Both peptides exhibited strong bactericidal activity, causing membrane disruption, and effectively interfered with biofilm development, including inhibition of initial attachment, biofilm maturation, and reduction in established biomass. Additionally, they suppressed the expression of key virulence genes associated with biofilm formation, while maintaining low cytotoxicity toward human keratinocytes.

Taken together, pantinin-1 and pantinin-2 are promising candidates for the development of novel anti-infective strategies against MDR *A. baumannii*. Further studies are needed to evaluate their activity against a broader panel of clinical isolates, in vivo efficacy, optimal formulation and delivery, and long-term stability. These findings provide a strong foundation for preclinical development and support ongoing efforts to identify innovative therapeutics targeting WHO-priority pathogens.

## Figures and Tables

**Table 1:** Sequence of the primers used for the PCR assays.

Gene	Forward Sequence (5′ → 3′)	Reverse Sequence (5′ → 3′)
*bap*	GTGGCTTAGACCGTTCACCA	CGAATCGAGCGCACAAGTTC
*smpA*	TGCAAAAACTCGTGCTGACG	GGGGATCAGTCACTGTTGGG
*pgaA*	TTGTCAGCAATTGTGTCGCA	ACCATCTTCCCCTGCATCAA
*16s*	GGTAGAGTTTGATCCTGGCTCAG	ATTACCGCGGCTGCTGG

## Data Availability

The original contributions presented in this study are included in the article/Supplementary Materials. Further inquiries can be directed to the corresponding author.

## Supplementary Materials

The following supporting information can be downloaded at: https://www.mdpi.com/article/10.3390/microorganisms14010068/s1, Table S1. Antibiogram *A. baumannii* 2403 isolated from peripheral vein blood culture. The strain producing class D carbapenemase (oxa-48) resulted positive after 6 h. Figure S1. Evaluation of anti-biofilm activity of pantinins against *A. baumannii.* (A) Impact of pantinin-1 (P1) in inhibiting *A. baumannii* biofilm and (B) degradation. (C) Effect of pantinin-2 (P2) during attachment phase. CTR−: untreated bacteria. Data represent the mean ± SD. Statistical significance was determined using Dunnett’s multiple comparison test: ns = not significant. Figure S2. SEM analysis of *A. baumannii* cells treated with different concentrations of pantinins. Cells were exposed to 2× MIC (A,D), 1× MIC (B,E), and ½× MIC (C,F) of each peptide. CTR− corresponds to untreated bacteria (H), while CTR+ corresponds to bacteria treated with gentamicin (4 μg/mL) (G). Images were acquired at a magnification of 10,000×.

## Abbreviations

The following abbreviations are used in this manuscript:

*A. baumannii*	*Acinetobacter baumannii*
AMPs	Antimicrobial Peptides
MIC	Minimum Inhibitory Concentration
MBC	Minimal Bactericidal Concentration
SEM	Scanning Electron Microscopy
CV	Crystal Violet
MTT	3-(4,5-dimethylthiazol-2-yl)-2,5-diphenyltetrazolium bromide
HaCaT	Human Keratinocyte Cell Line
ATCC	American Type Culture Collection
DMSO	Dimethyl Sulphoxide
CTR+	Positive Control
CTR−	Negative Control
DMEM	Dulbecco’s Modified Eagle Medium
FBS	Bovine Fetal Serum
MH	Müller–Hinton
LB	Luria–Bertani
MALDI-TOF	Matrix-Assisted Laser Desorption/Ionization–Time of Flight
EUCAST	European Committee on Antimicrobial Susceptibility Testing
CFU	Colony-Forming Unit
PBS	Phosphate-Buffered Saline
O/N	Overnight
PCR	Polymerase Chain Reaction
cDNA	Complementary DNA
RT	Reverse Transcription
SD	Standard Deviation
ANOVA	Analysis of Variance
CRAB	Carbapenem-Resistant Acinetobacter baumannii
MDR	Multidrug-Resistant
XDR	Extensively Drug-Resistant
PDR	Pan-Drug-Resistant
VAP	Ventilator-Associated Pneumonia
PNAG	Poly-β-(1-6)-N-acetylglucosamine
ST	Sequence Type
