# Supplementary material for: Scorpion Venom-Derived Peptides: A New Weapon Against Carbapenem-Resistant Acinetobacter baumannii
Source: Microorganisms. 2025 Dec 28;14(1):68. doi: 10.3390/microorganisms14010068 (PMC12844211; doi:10.3390/microorganisms14010068)
Supplement: Supplementary file 1 [file microorganisms-14-00068-s001.zip › microorganisms-4029615-supplementary.pdf]

Table S1. Antibiogram *A. baumannii* 2403 isolated from peripheral vein blood culture. The strain producing class D carbapenemase (oxa-48) resulted positive after 6 hours.

Active agent	MIC	SRI
Amikacin	>16	(R)
Ciprofloxacin	>1	(R)
Colistin	<=1	(S)
Gentamicin	>4	(R)
Imipenem	>8	(R)
Levofloxacin	>1	(R)
Meropenem	>16	(R)
Tobramycin	>4	(R)
Trimethoprim-sulfamethoxazole	>4/76	(R)
Cefiderocol		(S)

Key: S = sensitive; I = intermediate; R = resistant; MIC = Minimum Inhibitory Concentration (μg/mL). The chemoantibiotic sensitivity has been determined by following the EUCAST guidelines 2024.

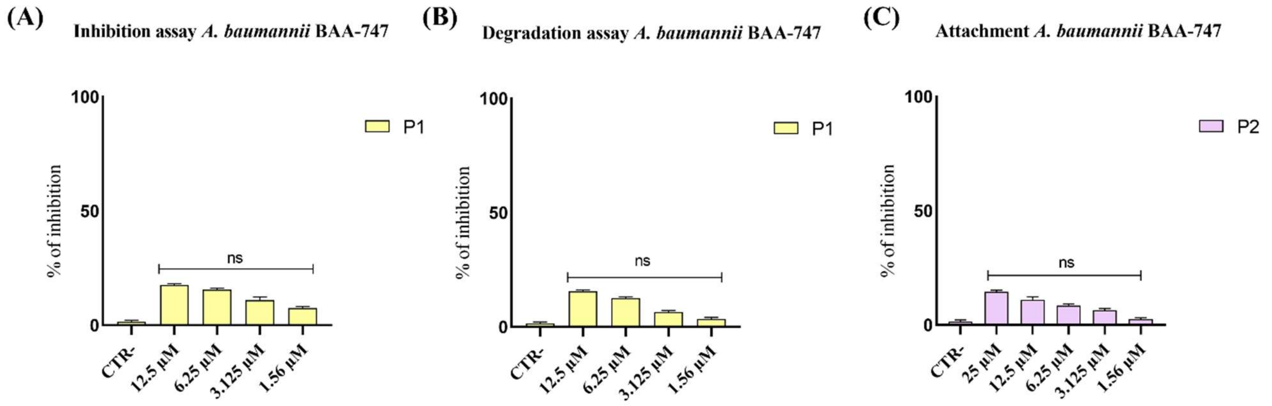

Figure S1. Evaluation of anti-biofilm activity of pantinins against *A. baumannii.* (A) Impact of pantinin-1 (P1) in inhibiting *A. baumannii* biofilm and (B) degradation. (C) Effect of pantinin-2 (P2) during attachment phase. CTR −: untreated bacteria. Data represent the mean ± SD. Statistical significance was determined using Dunnett's multiple comparison test: ns = not significant.

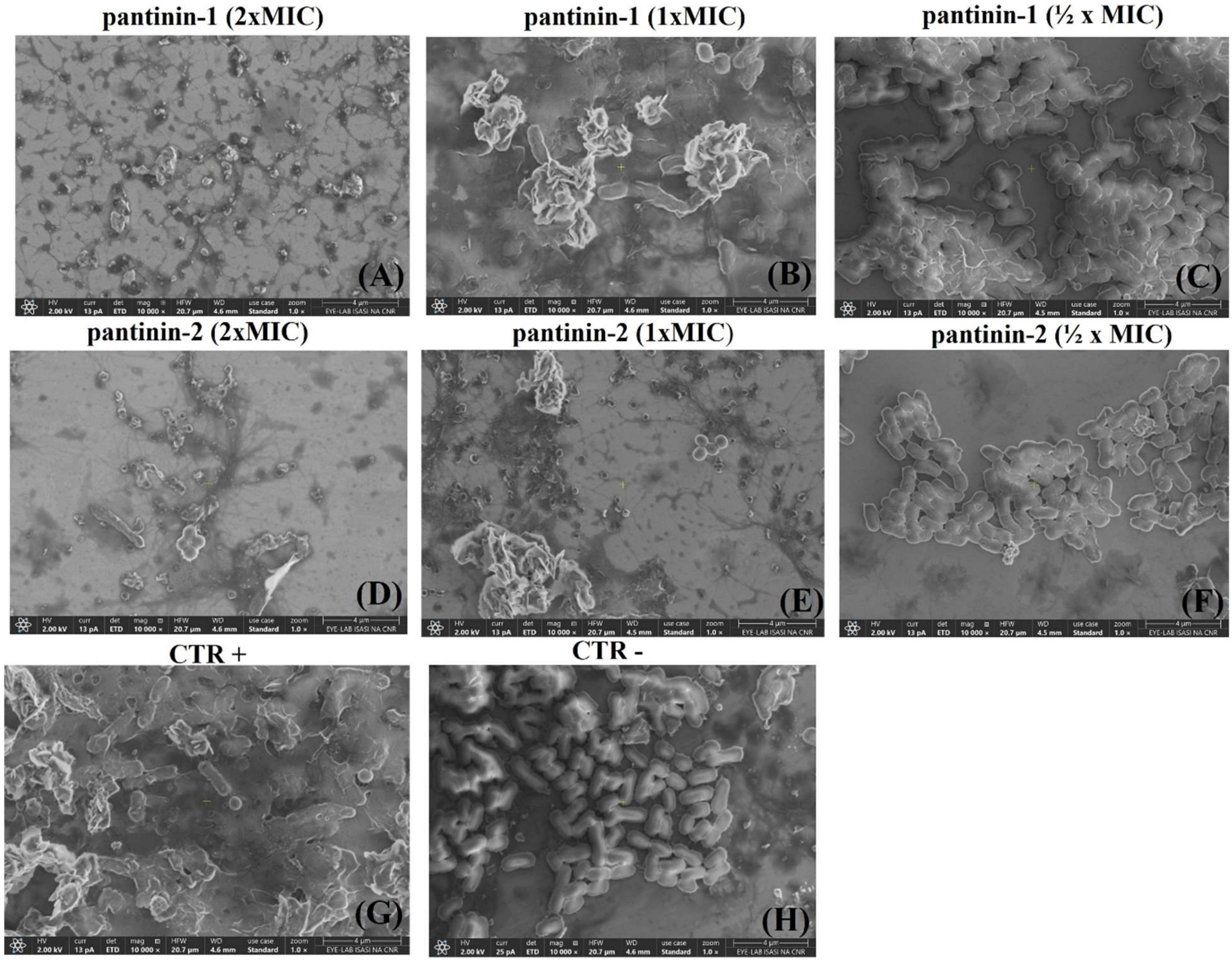

Figure S2. SEM analysis of *A. baumannii* cells treated with different concentrations of pantinins. Cells were exposed to 2× MIC (A–D), 1× MIC (B–E), and ½× MIC (C–F) of each peptide. CTR− corresponds to untreated bacteria (H), while CTR+ corresponds to bacteria treated with gentamicin (4 μg/mL) (G). Images were acquired at a magnification of 10,000×.
